# Gut Microbiota, Microbial Metabolites, and Inflammation in Cardiac Surgery: Implications for Clinical Outcomes—A Narrative Review

**DOI:** 10.3390/microorganisms13081748

**Published:** 2025-07-26

**Authors:** Panagiota Misokalou, Arezina N. Kasti, Konstantinos Katsas, Dimitrios C. Angouras

**Affiliations:** 1 Department of Nutrition and Dietetics, Attikon University General Hospital, 12462 Athens, Greece; gmisokalou@gmail.com (P.M.); kastiare@med.uoa.gr (A.N.K.); katkonstantinos@gmail.com (K.K.); 2 Medical School, National and Kapodistrian University of Athens, 75 Mikras Asias Street, 11527 Athens, Greece; 3 Department of Cardiac Surgery, School of Medicine, Attikon University Hospital, National and Kapodistrian University of Athens, 12462 Athens, Greece

**Keywords:** gut microbiota, dysbiosis, cardiac surgery, inflammation, cardiopulmonary bypass, postoperative atrial fibrillation

## Abstract

Cardiac surgery, particularly procedures involving cardiopulmonary bypass (CPB), is associated with a high risk of postoperative complications, including systemic inflammatory response syndrome (SIRS), postoperative atrial fibrillation (POAF), and infection. Growing evidence suggests that the gut–heart axis, through mechanisms involving intestinal barrier integrity and gut microbiota homeostasis, may influence these outcomes. This review summarizes the relationship between gut microbiota composition and the inflammatory response in patients undergoing cardiac surgery and the extent to which these alterations impact clinical outcomes. The reviewed studies consistently show that cardiac surgery induces notable alterations in microbial diversity and composition during the perioperative period. These changes, indicative of dysbiosis, are characterized by a reduction in health-associated bacteria such as *Blautia*, *Faecalibacterium*, and *Bifidobacterium* and an increase in opportunistic pathogens. Inflammatory biomarkers were frequently elevated postoperatively, even in patients without evident complications. Key microbial metabolites and biomarkers, including short-chain fatty acids (SCFAs), trimethylamine N-oxide (TMAO), and bile acids (BAs), were implicated in modulating inflammation and clinical outcomes. Additionally, vitamin D deficiency emerged as a contributing factor, correlating with increased systemic inflammation and a higher incidence of POAF. The findings suggest that gut microbiota composition prior to surgery may influence the severity of the postoperative inflammatory response and that perioperative modulation of the gut microbiota could represent a novel approach to improving surgical outcomes. However, the relationship between dysbiosis and acute illness in surgical patients is confounded by factors such as antibiotic use and other perioperative interventions. Large-scale, standardized clinical studies are needed to better define these interactions and guide future therapeutic strategies in cardiac surgery.

## 1. Introduction

The relationship between the gut microbiota and various organ systems has gained increasing attention in recent years. The human gut hosts trillions of symbiotic microorganisms, including bacteria, archaea, viruses, and fungi, collectively forming a complex microbial ecosystem known as the gut microbiota. This microbial community plays a crucial role in host metabolism, immune system modulation, energy homeostasis, vitamin synthesis, toxin elimination, and carbohydrate digestion [1,2]. Under normal conditions, a delicate balance is maintained between beneficial and pathogenic microbes, ensuring microbial homeostasis. However, when this balance is disrupted, a state known as gut dysbiosis emerges [3]. Characterized by a reduced abundance of the phyla *Bacteroidota* and *Bacillota* and an increased prevalence of *Pseudomonadota*, dysbiosis fosters the overgrowth of potentially pathogenic bacteria such as *Escherichia coli* and *Klebsiella* spp., both of which are frequently implicated in postoperative infections [4].

Several factors, including antibiotic use, Western-type dietary patterns, and lifestyle choices, can significantly influence the composition and function of the gut microbiota. Over the past decades, excessive caloric intake has disrupted lipid and carbohydrate metabolism, contributing to the onset and progression of cardiovascular diseases (CVDs). Emerging evidence highlights the significant role of gut dysbiosis in the pathophysiology of various CVDs, including atherosclerosis and heart failure, as well as metabolic disorders such as obesity, type 2 diabetes, and metabolic dysfunction-associated steatohepatitis. The concept of a gut–heart axis has recently gained traction, challenging the traditional view of the gut as an isolated organ. This bidirectional network underscores the importance of intestinal barrier integrity and microbial homeostasis in maintaining immune and metabolic balance [1]. Although it remains unclear whether gut dysbiosis is a cause or a consequence of postoperative complications, its association with systemic inflammation and bacterial translocation is well-documented [4].

While all surgical procedures elicit some degree of postoperative inflammation, cardiac surgery is particularly notable due to several additional contributing factors beyond surgical trauma. These include cardiopulmonary bypass (CPB), tissue hypoperfusion, and ischemia-reperfusion injury [5]. The acute-phase reaction following cardiac surgery triggers a surge in pro-inflammatory cytokines, including tumor necrosis factor-alpha (TNF-α) (which peaks immediately postoperatively), interleukin (IL)-6 and IL-8 (which peak later), and various chemokines that drive monocyte and macrophage activation [5,6,7]. This heightened inflammatory response can lead to systemic inflammatory response syndrome (SIRS), defined by the presence of at least two of the following criteria: (a) body temperature > 38 °C or <36 °C, (b) heart rate > 90 beats per minute, (c) respiratory rate > 20 breaths per minute or hyperventilation (PaCO_2_ < 4.3 kPa), and (d) leukocyte count abnormalities (>12,000/µL or <4000/µL, or >10% immature neutrophils) [8].

Postoperative systemic inflammation can have significant clinical implications, particularly in frail patients with substantial comorbidities. Infectious complications occur in 3.5% to 26.8% of cases and are associated with poorer outcomes [9,10]. Given these risks, monitoring and modulating inflammatory biomarkers have become key targets for developing preventive and therapeutic strategies [11].

Given the intricate interplay between gut microbiota and immune function, a strong link is expected between gut microbiota alterations and postoperative inflammatory or infectious complications following cardiac surgery. However, current evidence in this field remains sparse. This systematically informed narrative review synthesizes current evidence on the interplay between gut microbiota dysbiosis, microbial metabolites [short-chain fatty acids (SCFAs), trimethylamine N-oxide (TMAO), and bile acids (BAs)], and systemic inflammation in patients undergoing cardiac surgery, with a focus on clinical outcomes such as SIRS and postoperative atrial fibrillation (POAF).

We critically evaluate mechanistic pathways, perioperative risk factors (e.g., CPB, antibiotics), and potential therapeutic strategies to modulate the gut–heart axis.

## 2. Gut Microbiota and Inflammation

The gut microbiota interacts with the host primarily through the intestinal mucosal surface. A well-balanced microbial community is essential for maintaining the integrity of the intestinal epithelial barrier, a function achieved by preserving the structure of tight junction proteins (TJPs), upregulating mucin gene expression, and preventing the adhesion of pathogenic bacteria to epithelial cells [12]. Gut microbiota regulates the inflammatory response through two primary mechanisms:

a. Immune activation: disruptions in microbial homeostasis stimulate inflammatory responses in damaged organs by activating immune cells and promoting the release of pro-inflammatory cytokines.

b. Metabolite-driven inflammation: an imbalanced gut microbiota produces abnormal metabolites that influence the phenotype and function of immune cells, further amplifying the inflammatory reaction [13].

In the context of cardiac dysfunction, intestinal wall edema can lead to reduced intestinal blood flow, thereby compromising the structural integrity of the mucosal epithelial barrier and increasing permeability. As a result, bacterial translocation, along with the release of endotoxins and microbial metabolites into systemic circulation, may trigger a systemic inflammatory response [12].

Under normal conditions, gut microbiota composition remains relatively stable, with *Bacillota* and *Bacteroidota* accounting for approximately 90% of the microbial population, although their relative abundance varies among individuals. The remaining 10% consists of *Verrucomicrobiota*, *Actinomycetota*, *Pseudomonadota* (particularly from the Enterobacteriaceae family), and *Fusobacteriota*. Gut dysbiosis is characterized by reduced microbial diversity and an increased presence of pro-inflammatory species [3]. In CVDs, distinct microbial alterations have been observed: (a) patients with coronary artery disease (CAD) exhibit an overrepresentation of *Collinsella aerofaciens*, *Enterococcus*, *Megamonas*, and *Megasphaera*; (b) in valvular heart disease, microbial signatures are marked by *Bacteroides plebeius*, Enterobacteriaceae, *Veillonella dispar*, *Prevotella copri*, and *Fusobacteriota* [14]; and (c) patients at high risk for stroke show elevated levels of bacteria from the Enterobacteriaceae and Veillonellaceae families [15].

These findings underscore the potential role of gut microbiota in modulating systemic inflammation and influencing cardiovascular health.

### Bacterial Species and Metabolites Implicated in CVDs

Microbial sequencing analyses have revealed a wealth of information regarding the composition and functional role of gut microbiota in CVDs. Specific bacterial species and their metabolites, including SCFAs, trimethylamine (TMA), secondary BAs, phenylacetylglutamine (PAGln), lipopolysaccharide (LPS), and coprostanol, have been implicated in the development and progression of cardiovascular pathology [16]. SCFAs, primarily acetate, propionate, and butyrate, are produced through the fermentation of non-digestible carbohydrates in the large intestine [17]. These molecules differ in their absorption and metabolic pathways, as SCFAs diffuse directly into intestinal cells, enter the portal circulation, and reach systemic circulation via the liver [18]. Acetate contributes to lipogenesis and is preferentially oxidized in peripheral muscle, while propionate influences metabolic regulation by stimulating the release of peptide YY (PYY) and glucagon-like peptide 1 (GLP-1) and is primarily utilized in the liver for oxidation or gluconeogenesis. Butyrate, on the other hand, is metabolized within the gut epithelium, where it plays a critical role in maintaining gut barrier integrity and modulating inflammation. Additionally, acetate and propionate influence blood pressure regulation through a complex interplay involving the renin–angiotensin system and signaling via free fatty acid receptor 3 (FFAR3) [17].

Among bacterial-derived pro-inflammatory factors, LPS, a component of the outer membrane of Gram-negative bacteria, enters the systemic circulation upon bacterial cell death and elicits a robust inflammatory response, particularly in conditions associated with increased intestinal permeability. Elevated LPS levels have been observed in individuals with CAD, heart failure [19], and atherosclerosis, where it contributes to foam cell formation and cholesteryl ester accumulation from native low-density lipoproteins [16]. LPS interacts with toll-like receptors (TLRs), particularly TLR4 on endothelial cells, activating the myeloid differentiation primary response 88 (MYD88) pathway and promoting the expression of NOD-like receptors (NLRs), including the pyrin domain-containing protein 3 (NLRP3) inflammasome. This cascade triggers nuclear factor kappa-B (NF-κB) and mitogen-activated protein kinase (MAPK) signaling, leading to the production of key pro-inflammatory cytokines such as IL-6, IL-1, and TNF-α [19,20]. The activation of TLR4 further stimulates nicotinamide adenine dinucleotide phosphate (NADPH) oxidase, increasing reactive oxygen species (ROS) production, which in turn inhibits endothelial nitric oxide synthase (eNOS) activity, thereby contributing to endothelial dysfunction, vascular inflammation, and hypertension. Similarly, peptidoglycan from bacterial cell walls binds to nucleotide-binding oligomerization domain-containing proteins 1 and 2 (NOD1 and NOD2), further propagating the inflammatory response and promoting atherosclerosis [20].

The microbial metabolite TMA, generated by specific bacterial species such as *Clostridium*, *Proteus*, and *Escherichia* [21], undergoes hepatic oxidation by flavin-monooxygenase-3 (FMO3) to form TMAO, which is subsequently released into the systemic circulation [16]. TMAO contributes to vascular endothelial dysfunction by activating nitric oxide dismutase and interacting with leucine-rich repeats (LRRs) and the NLRP3 inflammasome, leading to increased intracellular calcium flux and heightened platelet reactivity [20]. Elevated TMAO levels have been associated with enhanced platelet activation and thrombosis risk, as this metabolite influences platelet aggregation through multiple agonists, including adenosine diphosphate, thrombin, and collagen. Additionally, TMAO interacts with phospholipids in platelet membranes and enhances inositol 1,4,5-trisphosphate (IP3)-mediated intracellular calcium release, further amplifying platelet activation. These processes are linked to the release of inflammatory mediators such as the CD40 ligand, which exacerbates endothelial dysfunction [22].

BAs, which facilitate lipid emulsification and absorption, are another key group of gut microbiota-derived metabolites involved in cardiovascular pathology. Primary BAs undergo microbial conversion to secondary BAs, predominantly by *Bacillota* and *Actinomycetota* [21,23]. This conversion influences host metabolism through activation of the farnesoid X receptor (FXR) in enterocytes and adipocytes, a pathway implicated in inflammation and metabolic disturbances.

Additionally, PAGln, a microbial metabolite of phenylalanine, has been linked to atherosclerosis, myocardial infarction, and stroke by enhancing platelet adhesion and thrombus formation via interactions with G-protein-coupled receptors, including adrenergic receptors [24]. Lastly, some gut bacteria can convert cholesterol into coprostanol, a non-absorbable sterol excreted in feces. The presence of coprostanol-producing bacteria in stool samples has been associated with reduced fecal cholesterol levels, suggesting a potential role of gut microbiota in cholesterol homeostasis [25,26].

Collectively, these microbial metabolites highlight the intricate relationship between gut microbiota composition, systemic inflammation, and cardiovascular disease progression.

## 3. Inflammation Before and After Cardiac Surgery

Patients undergoing cardiac surgery often have a pre-existing chronic inflammatory state linked to cardiac disease and comorbidities. Atherosclerosis is largely an inflammatory process, with atherogenic diseases such as diabetes and dyslipidemia triggering a vascular inflammatory response that contributes to stable atherosclerotic plaque formation, while plaque instability and disruption can result from additional inflammatory stimuli [27]. The passage of LPS and TMAO into systemic circulation acts synergistically with traditional cardiovascular risk factors to accelerate atherosclerosis. TMAO promotes endothelial dysfunction and vascular inflammation by enhancing platelet hyperreactivity through increased calcium flux and sensitization of IP3 receptors in cardiomyocytes, thereby establishing a proarrhythmic substrate [22]. Concurrently, LPS translocation in heart failure exacerbates cachexia through sustained release of pro-inflammatory cytokines such as TNF-α and IL-6, which further perpetuates systemic inflammation and cardiac remodeling [28]. The clinical relevance of these mechanisms is underscored by the EPIC-Norfolk prospective population study, which demonstrated a robust association between elevated plasma TMAO levels and incident acute coronary syndrome, establishing TMAO as both a prognostic biomarker and potential therapeutic target in CVDs [29]. Immunologic activation during cardiac surgery leads to inflammation that is typically protective but can result in significant complications following CPB. Nonimmunologic activation may contribute to fluid shifts and microemboli, increasing capillary permeability, interstitial edema, and organ dysfunction. The precise relationship between CPB-induced inflammatory responses and adverse outcomes remains unclear, with several hypotheses proposed. One suggests that the balance between pro-inflammatory and anti-inflammatory cytokines correlates with multiorgan injury severity, while another posits that SIRS emerges from a complex interplay between cytokine upregulation and compensatory anti-inflammatory mechanisms, potentially leading to immunosuppression and increased infection risk. The multiple-hit hypothesis proposes that CPB primes certain immune cells, exaggerating responses to subsequent stimuli such as infection. Various inflammatory mediators, including components of the complement system, cytokines, adhesion molecules, and immune effectors, contribute to these responses [30]. Beyond CPB, other perioperative factors, including hypothermia, hemodilution, electrolyte imbalances, and pharmacologic agents, have been implicated in triggering inflammatory responses during cardiac surgery [27].

Several mechanisms contribute to systemic inflammation following cardiac surgery. Blood contact with the CPB circuit activates leukocytes and endothelial cells, while the release of the aortic cross-clamp induces ischemia-reperfusion injury, leading to oxidative tissue damage and ROS production. These oxidative processes result in functional and structural changes that create a pathophysiological substrate for POAF, with an incidence as high as 60%, particularly in patients undergoing valvular procedures [27,31]. The peak incidence of POAF coincides with elevated concentrations of inflammatory cytokines, including IL-6, IL-8, and TNF-α, along with increased levels of C-reactive protein (CRP), which has been associated with negative inotropic effects [32,33].

Gut barrier dysfunction may be both a cause and a consequence of the inflammatory response. The integrity of the normal gut barrier is maintained by epithelial cells, primarily enterocytes covering the villi of the small bowel mucosa, which are interconnected by TJP. Reduced mesenteric blood flow results in enterocyte injury and disruption of tight junctions, leading to increased intestinal permeability [33]. The presence of pro-inflammatory cytokines and an impaired gut barrier allows luminal antigens to penetrate tissue, resulting in intestinal and systemic inflammation [34,35,36,37]. Increased gut permeability facilitates the release of bacterial metabolites and endotoxins, including LPS and TMAO, from the intestinal lumen into circulation, contributing to the maintenance and amplification of SIRS [28]. Cardiac surgery significantly reduces mesenteric blood flow due to extracorporeal circulation and permissive hypotension, further exacerbating gut barrier dysfunction and systemic inflammation [33].

## 4. Microbial Metabolites in Cardiac Surgery: Mechanisms of Inflammation Modulation

The gut microbiota exerts profound effects on postoperative outcomes through three principal classes of metabolites that engage specific metabolic and inflammatory pathways. SCFAs are produced through *Faecalibacterium prausnitzii* and *Roseburia intestinalis*. These metabolites mediate their anti-inflammatory effects through multiple mechanisms [38]. Butyrate, the most biologically active SCFA, inhibits histone deacetylases in immune cells, leading to increased histone acetylation and subsequent upregulation of forkhead box P3 expression in regulatory T cells [39]. Simultaneously, SCFAs activate G protein-coupled receptors free fatty acid 2 (FFAR2) and FFAR3 on intestinal epithelial cells, triggering a signaling cascade that inhibits NF-κB translocation and reduces production of pro-inflammatory cytokines, including TNF-α and IL-6 [40]. At the gut barrier level, butyrate serves as the primary energy source for colonocytes, enhancing TJP expression [zonulin (ZO-1), occludin (OCLN)] and stimulating mucin-2 secretion from goblet cells, thereby maintaining intestinal barrier integrity [41].

In contrast, TMAO represents a pro-inflammatory metabolite generated through a two-step microbe–host pathway. Specific gut bacteria (*Clostridium sporogenes*, *Escherichia fergusonii*) metabolize dietary phosphatidylcholine and L-carnitine into TMA, which is subsequently oxidized by hepatic FMO3 to form TMAO [42]. This metabolite exerts its pathological effects through activation of the NLRP3 inflammasome complex in macrophages and endothelial cells. Mechanistically, TMAO induces ROS production via NADPH oxidase upregulation, leading to thioredoxin-interacting protein dissociation from thioredoxin and subsequent binding to NLRP3. This triggers caspase-1 activation and cleavage of pro-IL-1β into its active form [43]. Additionally, TMAO enhances platelet responsiveness through increased calcium release from the endoplasmic reticulum via IP3 receptor sensitization, creating a pro-thrombotic state [22].

BA metabolism represents a complex interplay between host and microbial biochemistry. Primary BAs (cholic acid, chenodeoxycholic acid) are synthesized in hepatocytes from cholesterol via the cytochrome P450-mediated classic and alternative pathways [44]. Gut bacteria, particularly members of the *Clostridium* and *Bacteroides* genera, transform these primary BA into secondary forms (deoxycholic acid, lithocholic acid) through deconjugation and 7α-dehydroxylation reactions. These microbially modified BAs serve as ligands for nuclear receptors, most notably the FXR and membrane-bound Takeda G protein-coupled receptor 5 (TGR5). FXR activation in enterocytes induces fibroblast growth factor 19 secretion, which suppresses hepatic BA synthesis via the fibroblast growth factor receptor 4 and β-Klotho complex. Simultaneously, TGR5 signaling in macrophages increases cyclic AMP production, inhibiting NLRP3 inflammasome assembly [45]. However, under conditions of dysbiosis, elevated secondary BAs can exert pro-inflammatory effects by inducing mitochondrial ROS production and activating the sphingosine-1-phosphate receptor 2 (S1PR2) pathway in endothelial cells [46].

## 5. Gut Barrier Dysfunction Biomarkers

Gut permeability is identified using a combination of circulating markers and functional tests. The most commonly used marker is LPS, while circulating anti-LPS antibodies, soluble LPS receptor (sCD14), LPS-binding protein (LBP), and d-lactate serve as additional indicators of gut barrier dysfunction. Elevated circulating concentrations of diamine oxidase have also been associated with increased gut permeability [47]. Intestinal fatty acid binding protein (I-FABP), a protein released into the bloodstream by enterocytes, rises significantly during cardiac surgery when mucosal injury occurs [33]. Additionally, alterations in the expression of TJP (OCLN, CLDN, ZO-1) or a reduction in the mucus layer further signal gut barrier dysfunction [47].

## 6. Literature Search Strategy

This review examines the relationship between gut microbiota composition and the intensity of the inflammatory response in patients undergoing cardiac surgery, as well as its clinical implications, particularly infection and POAF. To identify relevant studies, a comprehensive literature search was conducted using standard MeSH terms. The search, performed in Medline and the Cochrane Library, included the terms “gut microbiota AND cardiac surgery AND inflammation”, “gut metabolites AND cardiac surgery AND inflammation”, and “dysbiosis AND cardiac surgery AND inflammation”. This process yielded 125 publications in English (accessed on 15 January 2025).

**Figure 1:**
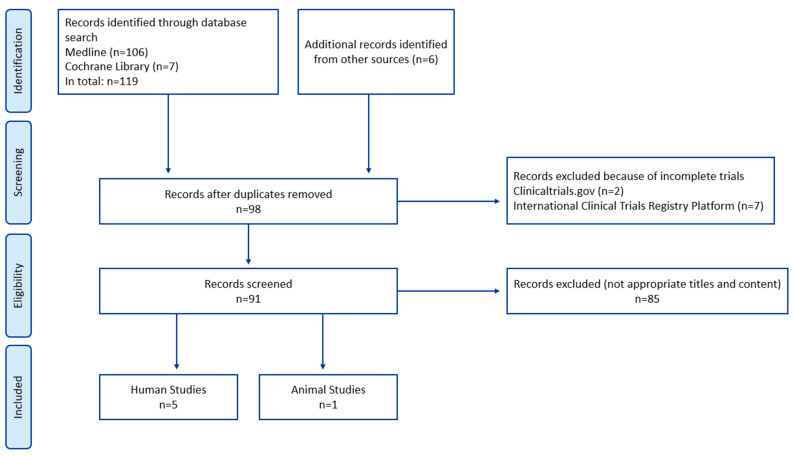
Flow chart. Identification and selection of the studies.

Only original research articles were considered, and additional studies were identified through manual searches of reference lists. The inclusion criteria encompassed studies on heart valve surgery, coronary artery bypass grafting (CABG), off-pump CABG, and combined procedures. Exclusion criteria included reviews, abstracts, conference presentations, editorials, and study protocols. Following an initial screening, 27 duplicate studies were removed, and 7 were excluded due to incomplete status. After further evaluation, 85 studies remained eligible for review. However, only six studies—five conducted in humans and one in animals—ultimately met the eligibility criteria, as summarized in Figure 1.

## 7. Summary of Studies Investigating Links Between Gut Microbiota, Cardiac Surgery, and Inflammation in Humans and Animals

Chernevskaya et al. analyzed gut microbiota composition before and after cardiac surgery to determine whether it serves as a marker for predicting bacterial infections. In this prospective pilot study, 72 patients were included, 12 of whom developed infectious complications. Inflammatory biomarkers and fecal samples were preoperatively and postoperatively assessed. The results demonstrated a significant shift in microbial composition in patients with infectious complications compared to those without, both before and after surgery. As expected, inflammatory biomarkers [IL-6 and high-sensitivity troponin (hs-TnT)] were significantly elevated in the infectious complications group postoperatively. The Shannon Index (α-diversity) was consistently low across all patients [48], consistent with prior studies linking reduced α-diversity to heart failure classification [49,50]. Interestingly, preoperatively, the Shannon Index was higher in the infectious complications group but decreased postoperatively, whereas it increased in patients without infections. This shift was attributed to an increase in *Pseudomonadota* in patients with infections. Notably, the study confirmed that patients who developed bacterial infections postoperatively already exhibited altered microbial compositions before surgery, reinforcing the authors’ hypothesis [48].

Liu et al. aimed to identify alterations in the fecal microbiome and plasma metabolome that distinguish patients with POAF from those without following CABG and to provide insights into POAF pathogenesis. Significant differences in gut microbiota diversity and composition were observed between POAF and non-POAF patients. Notably, TMAO levels were markedly higher in POAF patients. Additionally, both primary and secondary BA concentrations were significantly elevated in this group, with these elevations positively correlating with *Actinomycetota* and *Bacillota* abundance. This suggests a disruption in the gut microbiota–BA axis in POAF patients. Dysbiosis and aberrant BA metabolism, leading to elevated BA levels, can influence cardiac electrical activity and promote arrhythmogenesis. Furthermore, pro-BAs such as chenodeoxycholic acid can induce cardiac fibrosis via inflammatory pathways. The authors concluded that gut microbiota alterations and associated metabolic disturbances contribute to POAF onset and progression [23].

Maekawa et al., in a prospective observational study of 21 patients undergoing cardiac surgery with CPB, demonstrated that systemic inflammation and antibiotic administration led to a significant postoperative reduction in bacterial counts, altered gut microbiota composition, and increased fecal pH. Postoperatively, total bacterial populations and fecal SCFA concentrations were markedly reduced, while *Enterobacteriaceae*, *Enterococcus*, and *Staphylococcus* proliferated. Conversely, SCFA-secreting bacteria, such as *Lactobacillus*, declined, suggesting that gut microbiota disruption may contribute to leaky gut syndrome and bacterial translocation [51]. As previously discussed, bacterial translocation and increased gut permeability may perpetuate and exacerbate SIRS [33]. Although inflammatory markers were not directly measured in this study, the observed reduction in beneficial bacteria and SCFA levels post-surgery provides indirect evidence of dysbiosis, bacterial translocation, and inflammation.

Wang et al. investigated the relationship between gut microbiota composition and POAF. Fecal samples were collected from 45 patients with POAF and 90 matched non-POAF patients (1:2). Plasma 25-hydroxyvitamin D (25(OH)D) levels were also measured [52], given that vitamin D insufficiency or deficiency is associated with gut dysbiosis and increased susceptibility to inflammatory conditions [53,54]. The researchers hypothesized a potential link between vitamin D levels and POAF. They found that POAF patients exhibited significantly lower plasma 25(OH)D levels. Although postoperative serum inflammatory markers were not measured, differences in α- and β-diversities suggested that microbiota richness and diversity were significantly altered in POAF patients compared to non-POAF individuals, indicating a pre-existing microbial imbalance before POAF onset. At the genus level, POAF patients exhibited nearly twice the abundance of *Lachnospira* and reduced *Escherichia–Shigella* abundance compared to non-POAF patients [52].

Xia et al. examined whether perioperative alterations in gut microbiota are associated with systemic and intestinal inflammatory responses. The study assessed gut microbiota changes, intestinal homeostasis, and systemic inflammation before and after cardiovascular surgery in 67 patients. Postoperatively, biomarkers of gut barrier impairment (elevated IFABP and ZO-1) and local intestinal inflammation (increased lipocalin-2 and calprotectin) were significantly elevated. Microbiota analysis revealed a notable perioperative decline in α-diversity. Additionally, the postoperative gut microbiota was characterized by an increased abundance of *Enterococcus* and a decline in anaerobic, health-associated genera such as *Blautia*, *Faecalibacterium*, *Bifidobacterium*, *Roseburia*, *Gemmiger*, *Ruminococcus*, and *Coprococcus*. These microbial changes postoperatively correlated with elevated high-sensitivity CRP (hs-CRP), procalcitonin (PCT), TNF-α, and IL-6 levels. Collectively, these findings indicate that gut microbiota disturbances contribute to impaired intestinal homeostasis and systemic inflammation following cardiac surgery [37].

Salomon et al. assessed gut microbiota changes, intestinal barrier dysfunction, and inflammation-related metabolites in piglets subjected to cardiac surgery with CPB and deep hypothermic circulatory arrest (DHCA). A control group underwent mechanical ventilation without surgery. The CPB/DHCA group exhibited significantly elevated cytokine levels (IL-1β, IL-6, and TNF-α) compared to controls. Although α- and β-diversity analyses did not reveal significant differences between groups, α-diversity was reduced in samples collected before and after surgery. While the *Bacillota* and *Bacteroidota* phyla predominated in both groups, the CPB/DHCA group demonstrated a postoperative reduction in microbial species richness compared to preoperative samples. Although various animal models have been used to investigate CPB-related cardiovascular outcomes, this is the only study linking microbiome alterations, intestinal metabolites, and barrier dysfunction with post-CPB inflammation [55].

The main characteristics and findings of these studies are summarized in Table 1.

## 8. Discussion

Despite the relative scarcity of research in this area, the reviewed studies collectively demonstrate that cardiac surgery triggers significant alterations in gut microbiota composition, characterized by a reduction in beneficial bacteria such as *Faecalibacterium*, *Bifidobacterium*, and *Roseburia*, alongside an increase in opportunistic pathogens including *Enterococcus* and *Pseudomonadota*. These microbial shifts are associated with elevated levels of inflammatory markers, including TNF-α, IL-6, and CRP, and were linked to adverse clinical outcomes such as SIRS and POAF. The mechanisms underlying these associations involve the disruption of gut barrier integrity due to decreased SCFA production. The depletion of SCFA-producing genera (*Faecalibacterium*, *Roseburia*) disrupts gut barrier integrity through multiple mechanisms: butyrate deficiency reduces energy supply to colonocytes, downregulates TJP (ZO-1, OCLN), and impairs mucin-2 secretion from goblet cells [37,51]. This breach of intestinal barrier function permits translocation of pathogen-associated molecular patterns (PAMPs), with LPS activating TLR4/NF-κB signaling in endothelial cells and macrophages, thereby amplifying SIRS through IL-6 and TNF-α release [19,28,37].

Another relevant factor in this context is vitamin D, which plays a role in both inflammatory processes and gut microbiota composition [56]. Low circulating 25(OH)D levels are common before cardiac surgery and have been associated with an increased risk of major adverse cardiac events. Open-heart surgery itself acts as an acute stressor that further decreases circulating vitamin D concentrations, exacerbating pre-existing deficiencies [57]. The interplay between vitamin D status and gut microbiota may be particularly relevant in the pathogenesis of POAF, as low vitamin D levels have been linked to increased LPS levels, promoting inflammation [58] and potentially triggering atrial fibrillation [52]. Epidemiological studies have demonstrated an inverse relationship between vitamin D levels and inflammatory markers such as CRP and high-sensitivity CRP [59,60,61], suggesting that correcting vitamin D deficiency could mitigate systemic low-grade inflammation and potentially reduce the risk or severity of chronic inflammatory conditions [59]. Recent meta-analyses further support this association, confirming that vitamin D deficiency is a risk factor for POAF after CABG [62], while preoperative vitamin D supplementation in deficient or insufficient patients may reduce its incidence [63].

Although dysbiosis has been well established as a contributing factor in chronic diseases, its role in acute illness, particularly in surgical and critically ill patients, remains less clear. These patients are subjected to various medical interventions, including blood transfusions, parenteral nutrition, and multiple medications, all of which may influence gut microbiota composition [64]. Additionally, antibiotic prophylaxis with cefazolin, gentamicin, teicoplanin, or vancomycin is routinely administered in cardiac surgery [65,66], further complicating the assessment of dysbiosis. Cefazolin reduces microbial diversity while promoting the expansion of *Streptococcus* species, potentially increasing the risk of postoperative infections [67]. Similarly, vancomycin administration leads to a marked decline in beneficial genera from the *Lachnospiraceae* family, including *Roseburia*, *Coprococcus*, and *Cuminococcus*, accompanied by reduced α-diversity and altered β-diversity [68]. Teicoplanin induces a distinct shift in microbial populations, characterized by an increase in *Bacteroidales* and a decrease in *Clostridiales*, which may disrupt gut homeostasis [69]. Gentamicin, another frequently used antibiotic, causes significant structural changes in the jejunal microbiota, though its effects on α-diversity remain inconsistent across studies, with some reports contradicting the expected decrease in diversity [67]. The depletion of beneficial SCFA-producing bacteria (*Roseburia*, *Coprococcus*) and the overgrowth of opportunistic pathogens (*Streptococcus*) may exacerbate postoperative SIRS and POAF. Given these effects, optimizing antibiotic regimens to minimize microbiota disruption while maintaining efficacy against surgical pathogens represents a critical area for future research in cardiac surgery patients. Since antibiotics can significantly alter microbial diversity and taxonomic composition, their use represents a major confounding factor when evaluating the impact of gut microbiota alterations on postoperative outcomes [64].

In a prospective longitudinal study involving intensive care unit patients undergoing cardiac surgery, lower microbial α-diversity was associated with prolonged antibiotic exposure. Researchers observed substantial intra-individual variation in gut microbiota composition, with a marked shift during hospital admission, characterized by an increase in pathobionts and a concurrent decline in anaerobic gut bacteria beneficial to health. Although inflammatory markers were not directly measured in that study, the microbial alterations suggested an inflammatory state linked to dysbiosis [70].

The emerging understanding of the gut–heart axis reveals important parallels and distinctions among CVD entities, all of which share cardiometabolic syndrome as a common pathogenic foundation. When comparing CAD and heart failure [71], several key patterns emerge that highlight shared mechanisms and disease-specific microbiota signatures. In various CVDs, there are consistently higher levels of *Streptococcus* and *Streptococcaceae*, along with a decrease in *Faecalibacterium* and its species, *Faecalibacterium prausnitzii* [72]. In CAD patients, microbial profiling shows characteristic overrepresentation of *Collinsella aerofaciens* and *Enterococcus* [14]. This pattern differs markedly from the microbiota alterations seen in heart failure patients, where depletion of *Coriobacteriaceae*, *Erysipelotrichaceae*, and *Ruminococcaceae* was observed on the family level [49]. In valvular heart disease, microbial signatures are marked by *Bacteroides plebeius*, *Enterobacteriaceae*, *Veillonella dispar*, *Prevotella copri*, and *Fusobacteriota* [14], and patients at high risk for stroke show elevated levels of bacteria from the Enterobacteriaceae and Veillonellaceae families [15]. The postoperative cardiac surgery patients we reviewed demonstrate a unique dysbiosis profile, combining features of both CAD and heart failure, with acute reductions in *Blautia* and *Bifidobacterium* alongside blooms of *Enterococcus* and *Pseudomonadota* [37,48,51]. The metabolic consequences of these distinct dysbiosis patterns show important variations. While chronic CAD and heart failure exhibit gradual metabolic disturbances (e.g., persistent TMAO elevation), postoperative patients display an acute, dramatic metabolic shift characterized by simultaneous SCFA depletion and LPS increase [23,37,51]. This acute-on-chronic pattern may explain the particularly severe inflammatory responses seen after cardiac surgery compared to stable CVD patients. Notably, the BA metabolism alterations show disease-specific patterns. POAF patients demonstrate marked BA dysregulation [23], while chronic CAD shows nuanced BA changes [16]. This may reflect differential activation of the FXR receptor pathway across disease states.

The novelty of our findings lies in this comparative analysis, revealing cardiac surgery as a unique model combining elements of chronic CVD pathophysiology with acute dysbiosis. This perspective helps bridge the gap between chronic cardiometabolic disease and acute surgical stress responses, offering new opportunities for targeted interventions. Future research should explicitly compare microbiota and metabolite profiles across CVD states to identify disease-specific therapeutic targets while capitalizing on shared pathways like SCFA restoration or LPS neutralization [12,17,37]. These insights carry important clinical implications. The recognition of perioperative gut dysbiosis as a modifiable risk factor opens new avenues for preoperative risk stratification and personalized interventions. Emerging evidence suggests that preoperative microbiota profiles may serve as valuable predictors of postoperative complications, including infections and POAF. However, several challenges remain in translating these findings into clinical practice. The concurrent effects of antibiotic use and CPB-induced ischemia on gut microbiota complicate the interpretation of observational studies, highlighting the need for carefully designed mechanistic investigations. Promising therapeutic strategies currently under investigation include probiotic and prebiotic interventions to restore SCFA-producing bacteria, dietary modifications to reduce TMAO precursor availability, and vitamin D supplementation to support gut barrier function (Table 2).

While preliminary results are encouraging, these approaches require rigorous validation through randomized controlled trials before they can be widely implemented in clinical practice. Future studies should focus on identifying optimal timing, duration, and patient selection criteria for these interventions to maximize their clinical benefit [23,37,48,51,52,55].

A comparative analysis of recent reviews on gut microbiota in cardiac surgery reveals some distinctions with our findings. Paneri and Sevta broadly cover gut microbiota dysbiosis in cardiac surgery patients, but they lack specific conclusions, offering only general observations without critical synthesis or clinical recommendations. While it summarizes various studies, it does not resolve conflicting evidence or establish causal relationships, heavily relying on observational data [78]. Mc Loughlin and Hinchion lack direct evidence linking gut microbiome composition to post-cardiac surgery inflammation, heavily relying on hypotheses and indirect associations from unrelated fields. It overlooks key confounders like antibiotic use and patient comorbidities, which significantly impact microbiome dynamics [64]. Zhang et al. applied machine learning to predict complications like cardiac surgery-associated acute kidney injury using microbiota profiles but lacked a mechanistic exploration of POAF pathways and partially relied on retrospective data [79]. On the contrary, our review integrates three key pathways—SCFA depletion, TMAO elevation, and BA dysregulation—with evidence from both human studies and animal models, while explicitly addressing confounders like antibiotic use. Unlike previous works, we establish causal links (e.g., TMAO-NLRP3 activation-POAF) and propose novel therapeutic targets (e.g., FXR agonists), advancing the field from observational associations to actionable mechanistic insights. Our strict inclusion of prospective studies and stratification by surgical type further strengthens clinical applicability, addressing gaps in prior reviews while setting standards for future research on microbiota-targeted interventions in cardiac surgery.

Despite the insights, several limitations must be acknowledged. The primary limitation is the small number of studies available, which restricts the precision of the findings. Moreover, considerable heterogeneity exists across studies in terms of design, methodology, and patient populations, further complicating direct comparisons and the extraction of consistent conclusions. The limited sample sizes in many studies also reduce the statistical power of their findings. Additionally, the inclusion of both animal models and human studies presents inherent challenges. While animal models provide mechanistic insights, their findings may not always be translatable to human physiology due to species-specific differences. Conversely, human studies are often constrained by biases such as selection bias, confounding variables, and the challenges associated with small patient cohorts. These limitations underscore the need for large-scale, standardized research to clarify the clinical relevance of gut microbiota alterations in cardiac surgery patients. Given the routine use of antibiotic prophylaxis in this setting [65,66], future research should prioritize standardized protocols for microbiota analysis and perioperative biomarker assessment in larger patient cohorts. Key unanswered questions include whether dysbiosis is a cause or consequence of postoperative inflammation and whether preoperative microbiota profiling could guide personalized therapeutic interventions.

## 9. Conclusions

The findings from the reviewed studies suggest that cardiac surgery induces profound gut microbiota dysbiosis, characterized by depletion of SCFA-producing taxa (*Faecalibacterium*, *Roseburia*) and expansion of pathobionts (*Enterococcus*, *Pseudomonadota*). These shifts are not merely associative but mechanistically linked to adverse outcomes. For example, SCFA depletion compromises gut barrier integrity, permitting the translocation of LPS and other PAMPs that amplify systemic inflammation via TLR4/NF-κB signaling, thereby exacerbating SIRS [19,37]. TMAO overproduction promotes arrhythmogenesis through NLRP3 inflammasome activation in cardiomyocytes and IP3 receptor-mediated calcium dysregulation [22,31]. BA dysregulation drives fibrotic remodeling via S1PR2-dependent fibroblast activation, particularly in POAF [23,55]. Critically, preoperative microbial profiles predict postoperative complications—*Streptococcus* and *Blautia* abundance correlate with infection risk [48], while increased TMAO levels increase POAF likelihood [23]. This suggests dysbiosis is a modifiable risk factor. Modulating gut microbiota through dietary interventions, probiotics, or other microbiota-targeted therapies before surgery may help mitigate the risk of postoperative complications. However, despite growing interest in this field, research on the relationship between gut microbiota and postoperative outcomes in cardiac surgery remains limited. Given the SIRS triggered by CPB and its association with serious complications, further investigation is warranted to better understand the mechanistic links between gut microbiota and surgical outcomes. Future studies should focus on identifying effective perioperative interventions to optimize gut microbiota composition and potentially reduce postoperative inflammatory complications.

## Figures and Tables

**Table 1:** Summary of studies on cardiac surgery and gut microbiota in humans and animals. Gut microbiota findings include diversity indices (e.g., α-diversity, β-diversity) and taxonomic abundance at various levels (e.g., phylum, genus).

Author, Year	Study Design	Key Findings on Gut Microbiota	Metabolic Pathways/Key Metabolites	Inflammatory Biomarkers	Clinical Outcomes
Chernevskaya et al., 2021 [48]	Human (N = 72)	- Reduced α-diversity preoperatively in the infectious complications group. - Increased *Pseudomonadota* postoperatively in infections.	- Lower TMAO in the infectious complications group.	Elevated IL-6 and PCT postoperatively.	Infectious complications are linked to preoperative dysbiosis.
Wang et al., 2023 [52]	Human (N = 134)	- POAF patients: Higher α-diversity, increased *Lachnospira*, *Acinetobacter*; decreased *Escherichia–Shigella*.	- Vitamin D deficiency correlated with dysbiosis.	IL-6, CRP, and hs-TnT were preoperatively measured.	POAF is associated with gut microbiota imbalance.
Liu et al., 2024 [23]	Human (N = 90)	- POAF patients: Lower diversity, increased *Actinomycetota/Bacillota*. - Elevated *Roseburia*, *Coprococcus*.	- Increased TMAO, BAs, and SCFAs (acetic/propionic acid).	Not directly measured.	Dysbiosis and BA/TMAO are linked to POAF.
Xia et al., 2021 [37]	Human (N = 67)	- Postoperative decline in *Blautia*, *Faecalibacterium*; rise in *Enterococcus*. - Reduced α-diversity.	- Elevated LPS, sCD14, I-FABP (gut barrier dysfunction).	Increased hs-CRP, PCT, TNF-α, and IL-6 postoperatively.	Gut barrier dysfunction and systemic inflammation.
Maekawa et al., 2020 [51]	Human (N = 21)	- Postoperative reduction in *Clostridium*, *Lactobacillus*; rise in *Enterococcus*, *Staphylococcus*.	- Reduced butyric acid (SCFA).	Not measured.	Dysbiosis is linked to leaky gut and translocation.
Salomon et al., 2023 [55]	Animal (piglets, N = 12)	- Reduced α-diversity post-CPB. - Increased *Pseudomonadota*.	- Lower SCFAs; elevated FABP2, claudin-2/3 (gut barrier markers).	Elevated IL-1β, IL-6, and TNF-α postoperatively.	CPB-induced dysbiosis and inflammation.

Stool samples in Table 1 were collected preoperatively and on postoperative day one. Abbreviations: POAF, postoperative atrial fibrillation; CPB, cardiopulmonary bypass; hs-TnT, high-sensitivity troponin T; PCT, procalcitonin; hs-CRP, high-sensitivity C-reactive protein; TNF-α, tumor necrosis factor-alpha; IL-6, interleukin 6; FABP2, fatty acid-binding protein 2; sCD14, soluble CD14; SCFAs, short-chain fatty acids; I-FABP, intestinal fatty acid-binding protein; TMAO, trimethylamine N-oxide; CRP, C-reactive protein; BAs, bile acids; LPS, lipopolysaccharide.

**Table 2:** Therapeutic strategies targeting gut–heart axis in cardiac surgery patients.

Target	Strategy	Potential Benefit
SCFAs	Prebiotics (fiber), *Bifidobacterium* probiotics	Restore gut barrier integrity, reduce CRP/IL-6 levels
TMAO	Limit choline-rich foods (red meat), FMO3 inhibitors	Lower thrombosis risk, prevent POAF
BAs	FXR agonists (e.g., obeticholic acid)	Attenuate NLRP3 activation, improve endothelial function
Vitamin D	Preoperative supplementation in deficient patients	Reduce LPS translocation, maintain tight junctions, and decrease POAF incidence

Abbreviations: SCFAs, short-chain fatty acids; TMAO, trimethylamine N-oxide; BAs, bile acids; FXR, farnesoid X receptor; NLRP3, NOD-like receptor pyrin domain-containing 3; LPS, lipopolysaccharide; POAF, postoperative atrial fibrillation; CRP, C-reactive protein; FMO3, flavin-monooxygenase 3; IL-6, interleukin-6; [73,74,75,76,77].

## Data Availability

Not applicable.

## Abbreviations

BA	Bile acid
CABG	Coronary artery bypass grafting
CAD	Coronary artery disease
CLDN	Claudin
CPB	Cardiopulmonary bypass
CRP	C-reactive protein
CVDs	Cardiovascular diseases
DHCA	Deep hypothermic circulatory arrest
eNOS	Endothelial nitric oxide synthase
FFAR2	Free fatty acid receptor 2
FFAR3	Free fatty acid receptor 3
FMO3	Flavin-monooxygenase 3
FXR	Farnesoid X receptor
GLP-1	Glucagon-like peptide 1
hs-CRP	High-sensitivity C-reactive protein
hs-TnT	High-sensitivity troponin T
I-FABP	Intestinal fatty acid binding protein
IL	Interleukin
IP3	Inositol 1,4,5-trisphosphate
LBP	LPS-binding protein
LPS	Lipopolysaccharide
MAPK	Mitogen-activated protein kinase
MYD88	Myeloid differentiation primary response gene 88
NADPH	Nicotinamide adenine dinucleotide phosphate
NF-κB	Nuclear factor kappa-B
NLR/NLRP3	NOD-like receptors/pyrin domain-containing protein 3
NOD1/2	Nucleotide-binding oligomerization domain-containing proteins 1 and 2
OCLN	Occludin
PAGIn	Phenylacetylglutamine
PAMPs	Pathogen-associated molecular patterns
PCT	Procalcitonin
POAF	Postoperative atrial fibrillation
PYY	Peptide YY
ROS	Reactive oxygen species
sCD14	Soluble cluster of differentiation 14
SCFAs	Short-chain fatty acids
SIRS	Systemic inflammatory response syndrome
S1PR2	Sphingosine-1-phosphate receptor 2
TGR5	Takeda G protein-coupled receptor 5
TJPs	Tight junction proteins
TLRs	Toll-like receptors
TMA	Trimethylamine
TMAO	Trimethylamine N-oxide
TLR-4	Toll-like receptor-4
TNF-α	Tumor necrosis factor-alpha
ZO-1	Zonulin
25(OH)D	25-hydroxyvitamin D

## References

[1] Rivera K., Gonzalez L., Bravo L., Manjarres L., Andia M.E. (2024). The Gut-Heart Axis: Molecular Perspectives and Implications for Myocardial Infarction. Int. J. Mol. Sci..

[2] Zaher A., Elsaygh J., Peterson S.J., Weisberg I.S., Parikh M.A., Frishman W.H. (2024). The Interplay of Microbiome Dysbiosis and Cardiovascular Disease. Cardiol. Rev..

[3] Singh P., Meenatchi R., Ahmed Z.H.T., Thacharodi A., Rohinth M., Kumar R.R., Varthan H.M.K., Hassan S. (2024). Implications of the gut microbiome in cardiovascular diseases: Association of gut microbiome with cardiovascular diseases, therapeutic interventions and multi-omics approach for precision medicine. Med. Microecol..

[4] Spari D., Zwicky S.N., Yilmaz B., Salm L., Candinas D., Beldi G. (2023). Intestinal dysbiosis as an intraoperative predictor of septic complications: Evidence from human surgical cohorts and preclinical models of peritoneal sepsis. Sci. Rep..

[5] Viikinkoski E., Aittokallio J., Lehto J., Ollila H., Relander A., Vasankari T., Jalkanen J., Gunn J., Jalkanen S., Airaksinen J. (2024). Prolonged Systemic Inflammatory Response Syndrome After Cardiac Surgery. J. Cardiothorac. Vasc. Anesth..

[6] Kawamura T., Wakusawa R., Okada K., Inadat S. (1993). Elevation of cytokines during open heart surgery with cardiopulmonary bypass: Participation of interleukin 8 and 6 in reperfusion injury. Can. J. Anaesth..

[7] McBride W.T., Armstrong M.A., Crockard A.D., McMurray T.J., Rea J.M. (1995). Cytokine balance and immunosuppressive changes at cardiac surgery: Contrasting response between patients and isolated CPB circuits. Br. J. Anaesth..

[8] Squiccimarro E., Stasi A., Lorusso R., Paparella D. (2022). Narrative review of the systemic inflammatory reaction to cardiac surgery and cardiopulmonary bypass. Artif. Organs.

[9] Dabbagh A., Rajaei S., Monfared A.B., Keramatinia A.A., Omidi K. (2012). Cardiopulmonary bypass, inflammation and how to defy it: Focus on pharmacological interventions. Iran. J. Pharm. Res..

[10] Zukowska A., Zukowski M. (2022). Surgical Site Infection in Cardiac Surgery. J. Clin. Med..

[11] Aljure O.D., Fabbro M. (2019). Cardiopulmonary Bypass and Inflammation: The Hidden Enemy. J. Cardiothorac. Vasc. Anesth..

[12] Rahman M.M., Islam F., Or-Rashid M.H., Mamun A.A., Rahaman M.S., Islam M.M., Meem A.F.K., Sutradhar P.R., Mitra S., Mimi A.A. (2022). The Gut Microbiota (Microbiome) in Cardiovascular Disease and Its Therapeutic Regulation. Front. Cell. Infect. Microbiol..

[13] Wang W., Zhu L.J., Leng Y.Q., Wang Y.W., Shi T., Wang W.Z., Sun J.C. (2023). Inflammatory Response: A Crucial Way for Gut Microbes to Regulate Cardiovascular Diseases. Nutrients.

[14] Liu Z., Li J., Liu H., Tang Y., Zhan Q., Lai W., Ao L., Meng X., Ren H., Xu D. (2019). The intestinal microbiota associated with cardiac valve calcification differs from that of coronary artery disease. Atherosclerosis.

[15] Masenga S.K., Hamooya B., Hangoma J., Hayumbu V., Ertuglu L.A., Ishimwe J., Rahman S., Saleem M., Laffer C.L., Elijovich F. (2022). Recent advances in modulation of cardiovascular diseases by the gut microbiota. J. Hum. Hypertens..

[16] Nesci A., Carnuccio C., Ruggieri V., D’Alessandro A., Di Giorgio A., Santoro L., Gasbarrini A., Santoliquido A., Ponziani F.R. (2023). Gut Microbiota and Cardiovascular Disease: Evidence on the Metabolic and Inflammatory Background of a Complex Relationship. Int. J. Mol. Sci..

[17] Chambers E.S., Preston T., Frost G., Morrison D.J. (2018). Role of Gut Microbiota-Generated Short-Chain Fatty Acids in Metabolic and Cardiovascular Health. Curr. Nutr. Rep..

[18] Palm C.L., Nijholt K.T., Bakker B.M., Westenbrink B.D. (2022). Short-Chain Fatty Acids in the Metabolism of Heart Failure—Rethinking the Fat Stigma. Front. Cardiovasc. Med..

[19] Bowman J.D., Surani S., Horseman M.A. (2017). Endotoxin, Toll-like Receptor-4, and Atherosclerotic Heart Disease. Curr. Cardiol. Rev..

[20] Dicks L.M.T. (2024). Cardiovascular Disease May Be Triggered by Gut Microbiota, Microbial Metabolites, Gut Wall Reactions, and Inflammation. Int. J. Mol. Sci..

[21] Al Bander Z., Nitert M.D., Mousa A., Naderpoor N. (2020). The Gut Microbiota and Inflammation: An Overview. Int. J. Environ. Res. Public Health.

[22] Zhu W., Gregory J.C., Org E., Buffa J.A., Gupta N., Wang Z., Li L., Fu X., Wu Y., Mehrabian M. (2016). Gut Microbial Metabolite TMAO Enhances Platelet Hyperreactivity and Thrombosis Risk. Cell.

[23] Liu Y., Du Z., Lu Y., Ma Y., Yang Y., Osmanaj F., Zhang Y., Guo X., Qin Y., Yang X. (2024). Gut microbiota metabolism disturbance is associated with postoperative atrial fibrillation after coronary artery bypass grafting. Npj Cardiovasc. Health.

[24] Zhu Y., Dwidar M., Nemet I., Buffa J.A., Sangwan N., Li X.S., Anderson J.T., Romano K.A., Fu X., Funabashi M. (2023). Two distinct gut microbial pathways contribute to meta-organismal production of phenylacetylglutamine with links to cardiovascular disease. Cell Host Microbe.

[25] Kazemian N., Mahmoudi M., Halperin F., Wu J.C., Pakpour S. (2020). Gut microbiota and cardiovascular disease: Opportunities and challenges. Microbiome.

[26] Kenny D.J., Plichta D.R., Shungin D., Koppel N., Hall A.B., Fu B., Vasan R.S., Shaw S.Y., Vlamakis H., Balskus E.P. (2020). Cholesterol Metabolism by Uncultured Human Gut Bacteria Influences Host Cholesterol Level. Cell Host Microbe.

[27] Zakkar M., Ascione R., James A.F., Angelini G.D., Suleiman M.S. (2015). Inflammation, oxidative stress and postoperative atrial fibrillation in cardiac surgery. Pharmacol. Ther..

[28] Di Vincenzo F., Del Gaudio A., Petito V., Lopetuso L.R., Scaldaferri F. (2024). Gut microbiota, intestinal permeability, and systemic inflammation: A narrative review. Intern. Emerg. Med..

[29] Tang W.H.W., Li X.S., Wu Y., Wang Z., Khaw K.T., Wareham N.J., Nieuwdorp M., Boekholdt S.M., Hazen S.L. (2021). Plasma trimethylamine N-oxide (TMAO) levels predict future risk of coronary artery disease in apparently healthy individuals in the EPIC-Norfolk prospective population study. Am. Heart J..

[30] Banerjee D., Feng J., Sellke F.W. (2024). Strategies to attenuate maladaptive inflammatory response associated with cardiopulmonary bypass. Front Surg..

[31] Castillo R.L., Farías J., Sandoval C., González-Candia A., Figueroa E., Quezada M., Cruz G., Llanos P., Jorquera G., Kostin S. (2024). Role of NLRP3 Inflammasome in Heart Failure Patients Undergoing Cardiac Surgery as a Potential Determinant of Postoperative Atrial Fibrillation and Remodeling: Is SGLT2 Cotransporter Inhibition an Alternative for Cardioprotection?. Antioxidants.

[32] Gaudino M., Di Franco A., Rong L.Q., Piccini J., Mack M. (2023). Postoperative atrial fibrillation: From mechanisms to treatment. Eur. Heart J..

[33] Habes Q.L.M., Kant N., Beunders R., van Groenendael R., Gerretsen J., Kox M., Pickkers P. (2023). Relationships Between Systemic Inflammation, Intestinal Damage and Postoperative Organ Dysfunction in Adults Undergoing Low-Risk Cardiac Surgery. Heart Lung Circ..

[34] Lau L.L., Halliday M.I., Lee B., Hannon R.J., Gardiner K.R., Soong C.V. (2000). Intestinal Manipulation During Elective Aortic Aneurysm Surgery Leads to Portal Endotoxaemia and Mucosal Barrier Dysfunction. Eur. J. Vasc. Endovasc. Surg..

[35] Derikx J.P.M., van Waardenburg D.A., Thuijls G., Willigers H.M., Koenraads M., van Bijnen A.A., Heineman E., Poeze M., Ambergen T., van Ooij A. (2008). New Insight in Loss of Gut Barrier during Major Non-Abdominal Surgery. PLoS ONE.

[36] Kano H., Takahashi H., Inoue T., Tanaka H., Okita Y. (2017). Transition of intestinal fatty acid-binding protein on hypothermic circulatory arrest with cardiopulmonary bypass. Perfusion.

[37] Xia X., Ni J., Yin S., Yang Z., Jiang H., Wang C., Peng J., Wei H., Wang X. (2021). Elevated Systemic and Intestinal Inflammatory Response Are Associated with Gut Microbiome Disorder After Cardiovascular Surgery. Front. Microbiol..

[38] Parada Venegas D., De la Fuente M.K., Landskron G., González M.J., Quera R., Dijkstra G., Harmsen H.J.M., Faber K.N., Hermoso M.A. (2019). Short Chain Fatty Acids (SCFAs)-Mediated Gut Epithelial and Immune Regulation and Its Relevance for Inflammatory Bowel Diseases. Front. Immunol..

[39] Schulthess J., Pandey S., Capitani M., Rue-Albrecht K.C., Arnold I., Franchini F., Chomka A., Ilott N.E., Johnston D.G.W., Pires E. (2019). The Short Chain Fatty Acid Butyrate Imprints an Antimicrobial Program in Macrophages. Immunity.

[40] Li M., van Esch B.C.A.M., Wagenaar G.T.M., Garssen J., Folkerts G., Henricks P.A.J. (2018). Pro- and anti-inflammatory effects of short chain fatty acids on immune and endothelial cells. Eur. J. Pharmacol..

[41] Cao Y., Chen J., Xiao J., Hong Y., Xu K., Zhu Y. (2024). Butyrate: A bridge between intestinal flora and rheumatoid arthritis. Front. Immunol..

[42] Chan M.M., Yang X., Wang H., Saaoud F., Sun Y., Fong D. (2019). The Microbial Metabolite Trimethylamine N-Oxide Links Vascular Dysfunctions and the Autoimmune Disease Rheumatoid Arthritis. Nutrients.

[43] Sun X., Jiao X., Ma Y., Liu Y., Zhang L., He Y., Chen Y. (2016). Trimethylamine N-oxide induces inflammation and endothelial dysfunction in human umbilical vein endothelial cells via activating ROS-TXNIP-NLRP3 inflammasome. Biochem. Biophys. Res. Commun..

[44] Chen I., Cassaro S. (2023). Physiology, Bile Acids. *StatPearls [Internet]*. https://www.ncbi.nlm.nih.gov/books/NBK549765/.

[45] Larabi A.B., Masson H.L.P., Bäumler A.J. (2023). Bile acids as modulators of gut microbiota composition and function. Gut Microbes.

[46] Hylemon P.B., Su L., Zheng P., Bajaj J.S., Zhou H. (2021). Bile Acids, Gut Microbiome and the Road to Fatty Liver Disease. Comprehensive Physiology.

[47] Lewis C.V., Taylor W.R. (2020). Intestinal barrier dysfunction as a therapeutic target for cardiovascular disease. Am. J. Physiol.-Heart Circ. Physiol..

[48] Chernevskaya E., Zuev E., Odintsova V., Meglei A., Beloborodova N. (2021). Gut Microbiota as Early Predictor of Infectious Complications before Cardiac Surgery: A Prospective Pilot Study. J. Pers. Med..

[49] Luedde M., Winkler T., Heinsen F., Rühlemann M.C., Spehlmann M.E., Bajrovic A., Lieb W., Franke A., Ott S.J., Frey N. (2017). Heart failure is associated with depletion of core intestinal microbiota. ESC Heart Fail..

[50] Sun W., Du D., Fu T., Han Y., Li P., Ju H. (2022). Alterations of the Gut Microbiota in Patients with Severe Chronic Heart Failure. Front Microbiol..

[51] Maekawa M., Yoshitani K., Yahagi M., Asahara T., Shishido Y., Fukushima S., Tadokoro N., Fujita T., Ohnishi Y. (2020). Association between postoperative changes in the gut microbiota and pseudopsia after cardiac surgery: Prospective observational study. BMC Surg..

[52] Wang Y., He Y., Li R., Jiang H., Tao D., Zhao K., Yin Z., Zhang J., Wang H. (2023). Gut Microbiota in Patients with Postoperative Atrial Fibrillation Undergoing Off-Pump Coronary Bypass Graft Surgery. J. Clin. Med..

[53] Sukik A., Alalwani J., Ganji V. (2023). Vitamin D Gut Microbiota, and Cardiometabolic Diseases—A Possible Three-Way Axis. Int. J. Mol. Sci..

[54] Gong J., He L., Zou Q., Zhao Y., Zhang B., Xia R., Chen B., Cao M., Gong W., Lin L. (2022). Association of serum 25-hydroxyvitamin D (25(OH)D) levels with the gut microbiota and metabolites in postmenopausal women in China. Microb. Cell Fact..

[55] Salomon J.D., Qiu H., Feng D., Owens J., Khailova L., Osorio Lujan S., Iguidbashian J., Chhonker Y.S., Murry D.J., Riethoven J.J. (2023). Piglet cardiopulmonary bypass induces intestinal dysbiosis and barrier dysfunction associated with systemic inflammation. Dis. Model Mech..

[56] Bellerba F., Muzio V., Gnagnarella P., Facciotti F., Chiocca S., Bossi P., Cortinovis D., Chiaradonna F., Serrano D., Raimondi S. (2021). The Association between Vitamin D and Gut Microbiota: A Systematic Review of Human Studies. Nutrients.

[57] Barker T., May H.T., Doty J.R., Lappe D.L., Knowlton K.U., Carlquist J., Konery K., Inglet S., Chisum B., Galenko O. (2021). Vitamin D supplementation protects against reductions in plasma 25-hydroxyvitamin D induced by open-heart surgery: Assess-d trial. Physiol. Rep..

[58] Li Y., Si H., Ma Y., Li S., Gao L., Liu K., Liu X. (2023). Vitamin D3 affects the gut microbiota in an LPS-stimulated systemic inflammation mouse model. Microbes Infect..

[59] Zhou A., Hyppönen E. (2023). Vitamin D deficiency and C-reactive protein: A bidirectional Mendelian randomization study. Int. J. Epidemiol..

[60] Yang F., Sun M., Sun C., Li J., Yang X., Bi C., Wang M., Pu L., Wang J., Wang C. (2020). Associations of c-reactive protein with 25-hydroxyvitamin D in 24 Specific Diseases: A Cross-sectional Study from NHANES. Sci. Rep..

[61] Chen N., Wan Z., Han S.F., Li B.Y., Zhang Z.L., Qin L.Q. (2014). Effect of Vitamin D Supplementation on the Level of Circulating High-Sensitivity C-Reactive Protein: A Meta-Analysis of Randomized Controlled Trials. Nutrients.

[62] Hameed I., Malik S., Nusrat K., Siddiqui O.M., Khan M.O., Mahmood S., Memon A., Usman M.S., Siddiqi T.J. (2023). Effect of vitamin D on postoperative atrial fibrillation in patients who underwent coronary artery bypass grafting: A systematic review and meta-analysis. J. Cardiol..

[63] Ansari S.A., Dhaliwal J.S.S., Ansari Y., Ghosh S., Khan T.M.A. (2023). The Role of Vitamin D Supplementation Before Coronary Artery Bypass Grafting in Preventing Postoperative Atrial Fibrillation in Patients with Vitamin D Deficiency or Insufficiency: A Systematic Review and Meta-Analysis. Cureus.

[64] Mc Loughlin J., Hinchion J. (2023). The gut microbiome and cardiac surgery an unusual symphony. Perfusion.

[65] Cardiothoracic Interdisciplinary Research Network. Electronic address: CIRNetwork@outlook.com, National Cardiac Benchmarking Collaborative, Public Health England, Cardiothoracic Interdisciplinary Research Network (2020). National survey of variations in practice in the prevention of surgical site infections in adult cardiac surgery, United Kingdom and Republic of Ireland. J. Hosp. Infect..

[66] Edwards F.H., Engelman R.M., Houck P., Shahian D.M., Bridges C.R. (2006). The Society of Thoracic Surgeons Practice Guideline Series: Antibiotic Prophylaxis in Cardiac Surgery, Part I: Duration. Ann. Thorac. Surg..

[67] Zhang T., Wu X., Liu B., Huang H., Zhou C., Liang P. (2023). The contribution of probiotics for the double-edge effect of cefazolin on postoperative neurocognitive disorders by rebalancing the gut microbiota. Front. Neurosci..

[68] Nazzal L., Soiefer L., Chang M., Tamizuddin F., Schatoff D., Cofer L., Aguero-Rosenfeld M.E., Matalon A., Meijers B., Holzman R. (2021). Effect of Vancomycin on the Gut Microbiome and Plasma Concentrations of Gut-Derived Uremic Solutes. Kidney Int. Rep..

[69] Borsa B.A., Sudagidan M., Aldag M.E., Baris I.I., Acar E.E., Acuner C., Kavruk M., Ozalp V.C. (2021). Antibiotic administration in targeted nanoparticles protects the faecal microbiota of mice. RSC Med. Chem..

[70] Aardema H., Lisotto P., Kurilshikov A., Diepeveen J.R.J., Friedrich A.W., Sinha B., de Smet A.M., Harmsen H. (2020). Marked Changes in Gut Microbiota in Cardio-Surgical Intensive Care Patients: A Longitudinal Cohort Study. Front. Cell. Infect. Microbiol..

[71] von Bibra H., Paulus W., St. John Sutton M. (2016). Cardiometabolic Syndrome and Increased Risk of Heart Failure. Curr. Heart Fail. Rep..

[72] Martins D., Silva C., Ferreira A.C., Dourado S., Albuquerque A., Saraiva F., Batista A.B., Castro P., Leite-Moreira A., Barros A.S. (2024). Unravelling the Gut Microbiome Role in Cardiovascular Disease: A Systematic Review and a Meta-Analysis. Biomolecules.

[73] Pérez-Reytor D., Puebla C., Karahanian E., García K. (2021). Use of Short-Chain Fatty Acids for the Recovery of the Intestinal Epithelial Barrier Affected by Bacterial Toxins. Front Physiol..

[74] Jing L., Zhang H., Xiang Q., Shen L., Guo X., Zhai C., Hu H. (2022). Targeting Trimethylamine N-Oxide: A New Therapeutic Strategy for Alleviating Atherosclerosis. Front. Cardiovasc. Med..

[75] Han C. (2018). Update on FXR Biology: Promising Therapeutic Target?. Int. J. Mol. Sci..

[76] Cerit L., Özcem B., Cerit Z., Duygu H. (2018). Preventive Effect of Preoperative Vitamin D Supplementation on Postoperative Atrial Fibrillation. Braz. J. Cardiovasc. Surg..

[77] Lobo de Sá F.D., Backert S., Nattramilarasu P.K., Mousavi S., Sandle G.I., Bereswill S., Heimesaat M.M., Schulzke J.D., Bücker R. (2021). Vitamin D Reverses Disruption of Gut Epithelial Barrier Function Caused by Campylobacter jejuni. Int. J. Mol. Sci..

[78] Paneri M., Sevta P. (2022). Dysbiosis of Gut Microbiota in Patients Undergoing Cardiac Surgery. Glob. J. Med. Pharm. Biomed. Update.

[79] Zhang Y., Luo W., Zhao M., Li Y., Wu X. (2025). Advances in understanding the effects of cardiopulmonary bypass on gut microbiota during cardiac surgery. Int. J. Artif. Organs..

